# Feasibility and Acceptability of Intervention and Trial Procedures of the UCL Live Well With Parkinson's Self-Management Toolkit

**DOI:** 10.1155/padi/2804226

**Published:** 2025-08-21

**Authors:** Tasmin Rookes, Megan Armstrong, Kate Walters, Joy Read, Elizabeth Chesterman, Nathan Davies, Jennifer Pigott, Danielle Nimmons, Gareth Ambler, Mariam Adeleke, Rachael Hunter, Benjamin Gardner, Catherine Atkinson, Anette Schrag

**Affiliations:** ^1^Research Department of Primary Care and Population Health, University College London, London, UK; ^2^Department of Clinical and Movement Neurosciences, University College London, London, UK; ^3^Department of Statistical Science, University College London, London, UK; ^4^School of Psychology, University of Surrey, Guildford, UK; ^5^Homerton Healthcare NHS Foundation Trust, London, UK

## Abstract

Managing Parkinson's disease (PD) symptoms can be challenging due to multiple factors, including complex symptoms, which are often reported late, and a lack of resources, resulting in worse outcomes. Self-management of PD symptoms is a priority for patients, their carers, healthcare staff and systems. However, there is no effective comprehensive self-management intervention for use in the United Kingdom to support people with PD to self-manage problematic symptoms. We have developed a facilitated self-management toolkit through literature reviews and co-design workshops. We conducted a single-group, pre–post feasibility study to evaluate the feasibility and acceptability of this toolkit, ahead of a randomised controlled trial (RCT). We assessed the feasibility of the study by measuring recruitment rate, retention rate, data completion, outcome measures and serious adverse events. In addition, we collected fidelity data to ensure the intervention was delivered as designed. For acceptability, we measured participants' engagement through attendance at sessions, as well as through a feedback survey completed by participants at follow-up. In a subgroup of participants, we conducted semistructured interviews to gain feedback on what participants thought was good and what could be improved with the intervention, as well as how acceptable the trial procedures were. All quantitative data were summarised descriptively, and qualitative data were analysed using codebook thematic analysis. We successfully recruited the target population within a predefined timeline, maintained intervention engagement and completed sufficient follow-up, with limited missing data and no intervention-related serious adverse events. The intervention was delivered with 93% fidelity, and 89% of participants were engaged. Participants found the supporter sessions most helpful, followed by information pages, and setting person-centred goals. Having all their PD information in one place was seen as valuable, as well as talking through their challenges and problem-solving how to overcome them. The toolkit is now being tested in a national RCT.

**Trial Registration:** ISRCTN registry: ISRCTN92831552

## 1. Introduction

Parkinson's disease (PD) affects around 166,000 people in the United Kingdom and one in every 50 people over the age of 65 [1]. Both the number of people affected by PD and the associated costs are increasing due to the ageing population and are predicted to double by 2030 [2]. The associated disabling motor and non-motor symptoms result in an increased need for specialist and primary care services to provide support and, when the disease progresses, can result in hospitalisation [3]. Management of PD is challenging due to the complexity of symptoms, lack of resources and delayed reporting of symptoms [4, 5]. These complications underpin the 45% increased risk of hospital admission in people with PD compared to the general population, longer stays in hospital and in-patient deterioration, which takes longer to reverse after discharge [6].

Timely symptom management may be able to reduce complications and deterioration in PD. Self-management of symptoms and long-term conditions, like PD, are increasingly incorporated into healthcare systems to empower people and their carers and improve outcomes when access to services is becoming increasingly difficult. This approach of self-management is embedded in policy in the National Health Service (NHS) long-term plan [7]. Approaches to promote self-management include education, psychological support, strategies to adhere to treatment and tailored practical support [8], and there is evidence that these interventions are clinically effective, reducing healthcare utilisation without compromising patient outcomes [9].

Voluntary sector organisations, such as Parkinson's UK, offer information, advice and local self-management programmes and peer support groups [10]. In the United States, self-management interventions are being evaluated [11], but there is currently no effective comprehensive self-management intervention for use in the NHS in the United Kingdom to support people with PD and their carers to manage motor and non-motor symptoms. Current evidence for the effectiveness of self-management interventions for PD is mixed, with some randomised controlled trials (RCTs) showing benefits for physical functioning outcomes [12], but there are few large-scale high-quality studies [13]. Interventions often lack both the core components of self-management outlined in the self-management taxonomy and the perspective of people with PD [14–16]. In people with PD, self-efficacy and social support have consistently been highlighted as essential to improve self-management [16–18], and current digital interventions focus on symptom monitoring rather than symptom management, which is likely to have better clinical outcomes [19]. We have developed a self-management facilitated toolkit through synthesis of the self-management literature and co-design with people with PD, their carers and health and social care professionals [13, 14, 20, 21]. We aimed to develop a comprehensive intervention that supports personalised self-management in people with PD and their carers in managing motor and non-motor aspects of the condition, promoting wellbeing at all stages and being applicable in a range of different settings. We are reporting the results of a feasibility study of this new intervention to ensure the intervention is acceptable and that trial procedures are feasible for this population and to refine the intervention and study procedures.

## 2. Aim

To evaluate the feasibility and acceptability of study procedures and a facilitated self-management toolkit in people with PD and their carers, ahead of an RCT.

## 3. Methods

### 3.1. Study Design

A single-group, pre–post feasibility study with up to 35 participants, developed and reported in the context of with MRC framework for developing and evaluating complex interventions [22].

### 3.2. Participants and Procedures

People with PD living in the community, and their carers if relevant, were recruited from three secondary care sites and primary care in and around London for three months between October 2020 and January 2021. Sites screened their clinic lists against the inclusion and exclusion criteria and asked potentially eligible participants for consent to be approached by the study team. A member of the study team then contacted them to explain what the study involved and answered any questions they may have. Participants provided written or verbal consent to participate ahead of assessments and intervention delivery. This work is the main feasibility study from which a wearable device acceptability substudy was conducted [23].

#### 3.2.1. Inclusion Criteria

• Community-dwelling adults (i.e., 18 years and above)
• A confirmed diagnosis of PD (defined using UK Brain Bank Criteria [24]), including those with dementia diagnosed at least 1 year after their PD diagnosis.

#### 3.2.2. Exclusion Criteria

• A clinical diagnosis of atypical Parkinsonism
• Currently in hospital or living in a care home
• Lack of capacity to take part or a Telephone Montreal Cognitive Assessment (TMoCA) score of < 11 [25].
• Unable to engage in the intervention due to visual impairment or language barriers (and no carer or family member to support them to engage)
• Life expectancy < 6 months

### 3.3. Intervention

#### 3.3.1. UCL Live Well With Parkinson's Toolkit and Supporter Sessions

The intervention was a self-management intervention for community-dwelling people with PD, comprising of online and/or paper toolkit depending on the participant's preference and up to four sessions over 3 months with a trained ‘*Live Well*' supporter.

The intervention details have been reported elsewhere [20, 21]. In summary, participants and carers received access to an online and/or paper-based manual which contained information about symptoms, therapies/treatments, optimising wellbeing and practical advice. There were also seven personalised sections for self-completion covering information about themselves, their health and support, a calendar to track appointments, to-do lists and notes, the ability to review and track symptoms bothering them and an asset-based wellbeing section to identify health priorities and behaviours they wanted to maintain or improve and problem-solving to overcome barriers. The digital version was designed such that the personalised sections would link to the relevant information sections to facilitate more tailored information provision.

Alongside the toolkit, participants, and their carers if the participant wished, received up to four sessions over 3 months with the ‘*Live Well*' supporter (E.C.). The first two sessions were planned to last 60-90 minutes, followed by around 30 minutes for the remaining sessions. In these sessions, the supporter encouraged participants to self-manage their condition by using the ‘UCL *Live Well with Parkinson's'* toolkit, following a manual and checklists covering support navigating the toolkit, understanding the benefits of using the different sections and assistance with the creation of wellbeing priorities (goals) and use of behaviour change techniques to help implement priorities long-term. These sessions were conducted online via videoconference or telephone depending on participants' access to technology and their preference, due to COVID-19 restrictions. The toolkit contents, including component behaviour change techniques, are reported elsewhere, and the logic model can be found in Supporting Figure 1 [20, 21].

#### 3.3.2. The Supporter

The job description for the role was developed through the intervention co-design process [21] and finalised by the co-investigators to balance the desirable characteristics with cost and flexibility considerations for upscaling. The supporter was intended to be someone with a background in healthcare, social care, or third-sector care without specialist expertise in PD but with awareness of the problems people with PD may face. Qualities and skills of the supporter were prioritised for recruitment, whereas basic understanding of PD and Behaviour Change Theory were incorporated into training for the role. For this study, the supporter was a registered nurse with some generalist experience working with people with PD. The training plan was developed by the study team, including experts and people with lived experience of PD, behaviour change and self-management academics, and healthcare professionals.

The supporter received 6 days of training:

• Two days focussed on PD, including a free online training course from Parkinson's UK Excellence Network, a knowledge-focussed online training session delivered by J.P. (a clinical research fellow from a geriatric medicine background with expertise in PD) and in-person observations of an outpatient movement PD clinic.
• Two days of online training about the intervention manual and the toolkit, including a guided tour of the intervention delivered by J.P. (who led toolkit development), and M.A. (the programme manager), working through fictional case studies to demonstrate how the toolkit could be used during session delivery, and self-directed time exploring the toolkit resources.
• Two days of learning behaviour change and self-management techniques, developed and delivered by B.G. (a behaviour change expert who helped design the intervention). This training included how to support people to develop goals, build motivation, action planning, problem solving, providing feedback, coping with setbacks and forming habits.

The supporter received fortnightly online supervision of 30 minutes during the 3 months from M.A. (with expertise in behaviour change) and J.P. Supervisions were structured to cover general troubleshooting, debriefing challenges, discussing a case example, reflecting on past cases and identifying additional training needs. Additional support was provided by B.G. for specific challenges identified, and escalation procedures for potential issues (to A.S., a neurologist) were outlined but not needed.

### 3.4. Feasibility Criteria and Analysis

The criteria for successful feasibility of the study were the following:

• Recruitment rate of 35 people with PD over a three-month recruitment period
• Retention rate of 80% of participants completing the primary outcome at follow-up
• Minimum completion of 70% of all outcome measures (missingness)
• No serious intervention-related adverse events

Our main analysis was descriptive, focused on recruitment, engagement with the intervention, attrition from the research process and fidelity. Summary measures are presented as mean and standard deviation (SD) for continuous variables and frequencies and percentages for categorical variables for each measure pre- and post-test.

### 3.5. Trial Outcome Assessments

Measures were collected at baseline and 3-month follow-up (immediately postintervention). Due to COVID-19 restrictions, all assessments were conducted remotely via videoconference or telephone.

A full description of the outcome measures collected and analysed can be seen in the subsequent RCT protocol paper [20]. These included demographics, experiences of motor and non-motor symptoms (Movement Disorder Society–Unified Parkinson's Disease Rating Scale (MDS-UPDRS) [26] and Movement Disorder Society–Non-Motor Rating Scale (MDS-NMS) [27]), health status and quality of life (Parkinson's Disease Questionnaire-39 [28]), psychological wellbeing (General Health Questionnaire-12 [29]), patient activation (Patient Activation Measure-13 [30]) and self-efficacy [31]. Alongside clinical measures, an economic evaluation was conducted using three measures (ICEpop CAPability measure for Older people [32], EuroQol EQ-5D 5 level [EQ-5D-5L] [33] and Client Service Receipt Inventory-shortened, adapted for PD [34, 35]).

For this feasibility study, the primary outcome was the combined parts I and II of the MDS-UPDRS.

With participants who had a carer consented into the study, the carer also completed outcome measures focused on carer burden [36] and carer quality of life [37].

### 3.6. Fidelity of and Engagement With the Intervention

We assessed the fidelity of intervention delivery and participants' engagement with the intervention to ensure the intervention was delivered and received as intended, to support our understanding of the feasibility of delivering such an intervention and the intervention's acceptability in this population of people with PD.

Fidelity of intervention delivery was determined through session checklists based on the intervention manual. The checklists were completed at the end of each session by the supporter. If 80% or more of the compulsory components (28 aspects across four sessions) were delivered, the intervention was deemed to have fidelity. Additionally, 10% of the checklists were checked for reliability, with a member of the research team reading transcripts of sessions and completing the checklist independently. Data were also collected on participants' choice of toolkit format.

Engagement with the intervention was measured by the number of sessions participants attended out of four. Those who had engaged in three or four sessions were said to have engaged, those who attended two sessions were semiengaged and those who took part in one or no sessions were classed as having not engaged.

### 3.7. Acceptability of the Intervention, Outcome Measures and Research Process

All participants who completed follow-up assessments were invited to complete an additional survey, developed by the authors, measuring how helpful participants perceived the various toolkit sections, whether they set a goal and how much progress they felt they made, how helpful the supporter sessions were, whether they would recommend the toolkit and how they found the research processes. Free text boxes offered participants the option to add more detail about what they liked about the toolkit and sessions and how these could be improved.

Semistructured qualitative interviews were conducted to understand participants' experiences of how acceptable the intervention and research processes were. A subsample of participants, purposively sampled to ensure diversity of age, gender, ethnicity, education level, deprivation, disease severity, intervention engagement and toolkit type, were invited for interview. These were identified by creating a matrix of participant and toolkit characteristics. M.A., J.R. and T.R. then met to discuss which participants to sample to ensure a range of perspectives across the characteristics outlined above. All interviewers were female with an interest in self-management and behaviour change and experienced in qualitative research methods. All interviews were conducted remotely, via videoconferencing or telephone. Participants received a £20 voucher for taking part in the interview.

The topic guide was developed by the researchers (M.A., J.R., T.R., N.D., K.W. and A.S.) with input from public contributors with PD, their carers and the wider team. It was updated once, when half of the interviews had been completed, to probe on insights that were coming through and focus in on areas of interest that were not being covered. Conversations centred around feedback on toolkit content, sessions with the supporter, wellbeing and priority (goal) setting and progress, impact and implementation, and a review of study procedures. Interviews were audio-recorded and transcribed verbatim and anonymised by a third-party service. Transcripts were analysed using codebook thematic analysis [38], with the codebook developed by the researchers (M.A., T.R. and J.R.) after independently coding four of the transcripts and identifying common codes in the data. The remaining transcripts were coded in NVivo 11 by T.R.

### 3.8. Ethical Approval

This study was given a favourable opinion by the London Queen Square Research Ethics Committee and Health Research Authority approval (18/LO/1470) on 29 October 2018 and 31 October 2018, respectively. Ethical approval for the main trial was obtained separately on 18/10/2021 (21/LO/0562), after completion of this feasibility study.

## 4. Results

### 4.1. Participant Characteristics

Thirty-five participants were recruited, with a mean age of 69 years, 11 (31%) female, 26 (74%) White (British) and 18 (51%) living in the least deprived communities (Table 1). Of these 35 participants, 17 had a carer who consented alongside them.

### 4.2. Feasibility

#### 4.2.1. Recruitment Rate

Thirty-five participants, 100% of our target, were recruited over a 3-month period, meeting the feasibility criteria. In the first month, 13 participants were recruited, in the second month 9 participants, and in the third month 13 were recruited. We collected information on the number of people approached and how many of those were then screened and recruited. In total, 52 potential participants were approached, of which nine (17%) declined to participate and eight (15%) could not be contacted after they had expressed an interest and been sent the participant information sheet. Reasons for declining were being too unwell (*n* = 4), not thinking it would benefit them (*n* = 3), and not having the time to commit (*n* = 2).

#### 4.2.2. Retention Rate

**Figure 1:**
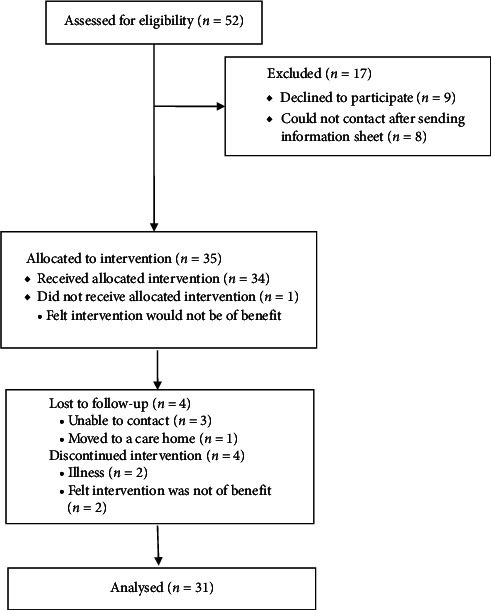
CONSORT 2010 flow diagram.

Thirty-one out of the 35 participants (89%) completed the primary outcome (parts I and II of MDS-UPDRS) at follow-up, surpassing the 80% feasibility criterion rate. Out of the 17 carers, 14 (78%) had complete follow-up data at 3 months. See Figure 1 for the recruitment flow diagram. Of the four participants who did not complete follow-up, all were White British males with an older mean age (73.5, SD = 3.9) than the overall sample. Three of these participants were using the paper toolkit, and one was using a combination of online and paper. Reasons for withdrawal were due to researchers being unable to contact the participants (*n* = 3) and the participant moving to a care home (*n* = 1).

#### 4.2.3. Missingness

All outcome measures had a response rate of above 70%, meeting the feasibility criteria. The MDS-UPDRS (parts I, II and IV), TMoCA, GHQ-12, PAM-13 and Zarit carer burden questionnaire had no missing items at baseline or follow-up. For the self-efficacy for managing chronic disease measure, there was one missing item at baseline for one participant and one missing item at follow-up for three participants. The PDQ-39 had one missing item for three participants at baseline and two at follow-up; two missing items by three participants at baseline and one at follow-up; and three missing items by one participant at baseline. The ICECAP-O had one missing item for two participants and two missing items for one participant at baseline. The MDS-UPDRS part III had missing data for all participants, as some elements of the assessment must be conducted face-to-face. Of assessments which could be collected, the MDS-NMS had the highest rate of missingness, with 17/35 participants at baseline missing one item and one participant at follow-up.

#### 4.2.4. Intervention-Related Adverse Events

No serious intervention-related adverse events were reported, again meeting the predetermined feasibility criterion.

### 4.3. Outcomes

Outcome data were available for all 35 participants at baseline and for 31 participants at 3-month follow-up.  Table 2 outlines the mean scores for all outcomes collected at baseline and 3-month follow-up. There were small, but not meaningful, reductions in MDS-UPDRS part I and part II. For the MDS-UPDRS part III, the number of missing items on the questionnaire, due to remote assessment, prevented calculation of a summary score.

### 4.4. Fidelity and Delivery

Of 35 participants, 22 participants used the online toolkit, 9 received the paper toolkit and 4 participants used a combination. The supporter delivered on average 93% of the 28 intervention components outlined in the intervention manual. This finding confirms the intervention was delivered as designed. The 10% checklists assessed by another member of the study team confirmed the fidelity percentage and highlighted areas of improvement in the checklists. For sessions three and four, it was challenging to differentiate between problem-solving and coping with setbacks. Occasionally, the supporter recorded problem-solving had occurred when the participant had not identified the solutions themselves. For two out of the 15 checklists checked, the researcher and supporter behaviour change technique ratings were noticeably different, with the supporter not documenting the techniques used.

### 4.5. Engagement

Of 35 participants, 31 (89%) engaged in the intervention attending at least 3 sessions, 3 (8%) semiengaged with the intervention attending 2 sessions, and 1 (3%) did not engage with any sessions. Reasons for lack of engagement were not feeling the intervention would benefit them (*n* = 2) and being too unwell (*n* = 2).

### 4.6. Acceptability

The feedback questionnaire was completed by 30/35 (86%) participants. Overall, participants found the toolkit and sessions to be helpful.  Table 3 highlights participants' responses to how helpful they found the individual toolkit components and the sessions. Of the 30 people who responded, 19 (63%) had set a wellbeing priority with the ‘*Live Well*' supporter, of which 12 felt they had made much progress towards this priority (63%). In terms of future use, 86% were likely or very likely to continue using the toolkit in the future and 87% were likely or very likely to recommend the toolkit to someone else with PD.

In terms of study procedures, 80% found the assessments easy to complete, 87% had no difficulty completing the questionnaires remotely, and 73% felt the assessment length was about right for what is expected in a research study. For a minority of participants, the assessments were seen as lengthy and difficult, especially when done remotely, and they had to be broken into numerous appointments.

Eighteen participants were invited to interview and 14 were interviewed, two of which were dyadic with carer and participant. Participant characteristics of those interviewed can be seen in Table 4. We were able to interview participants representative of the study population and purposively sampled those from more deprived backgrounds and who were younger. Four additional interviews were conducted with carers alone and one with the ‘*Live Well*' supporter, resulting in 19 total interviews. T.R. (*n* = 9), J.R. (*n* = 8) and M.A. (*n* = 2) conducted the interviews between March and May 2021. Three key themes were identified: (1) toolkit feedback, (2) changing or maintaining positive behaviours, and (3) context and complexity.

#### 4.6.1. Toolkit Feedback

Participants and carers found the toolkit useful to help self-manage symptoms, as it was comprehensive and gave a sense of empowerment in which they took some control over managing the disease. Having access to a paper version of the toolkit was also important for those with barriers using digital technology.

*I cannot tell you how positive I am about this kit… [and] what a useful tool it is. But how Parkinson's patients themselves should be enabled to do this, and I think it's fabulous, and it would be such a bonus.* Carer of Participant 34, Male 71 (gender and age are participants' demographics for all carer quotes)

The sessions were described as important by all participants to support navigating the toolkit, problem-solving and motivation. The supporter helped participants identify information that they found useful. There were no suggestions for improvement with the delivery of the sessions.

*I'd have been absolutely lost if I hadn't had that help at the outset. Some people, younger people are able to cope with computers and all that very easily, I wasn't able to.* Participant 15, Male 82

The person-centred approach to sessions and goals was deemed essential for this population with the need to adapt depending on their needs. Participants valued having someone to talk to, as they felt that this attention and time are often lacking in current healthcare systems.

Carers' interaction with the toolkit varied; some found it useful and reported that they used it more than the person with PD. Some carers allowed the participant to lead on the toolkit to promote autonomy, whereas some people with PD did not wish to share it with their carers, so as not to be defined by the condition.

*I'm conscious not to try and do things before [the person I care for] needs them done and thereby taking away some of her independence and liberty. So, I very much try and take the lead from her, so I don't think she felt I had to rush to the toolkit and get involved with it. If she felt that was helpful or that's what she wanted me to do, she would have told me.* Carer of Participant 12, Female 66

Participants did not share the toolkit with their HCPs, but many planned to do so in the future and felt that it would be helpful to do so.

The information sections were seen as comprehensive, easy to understand and trustworthy. Participants also found the signposting to additional resources useful.

*I liked the quotes from … other people [with Parkinson's] who've had ideas and suggestions. That's quite a human element of it that sort of makes you feel, “Oh, other people have been here before me, they've thought about this problem, this is their reaction.” And some great little tips as well.* Participant 24, Female 55

Although overall the information pages were reportedly helpful, some participants felt the amount of information provided was overwhelming. Participants wanted the information to be condensed, more personalised and presented in different ways to text only, such as videos.

*Yeah, there was too much information, I think. So, then you get more muddled.* Participant 19, Female 76

The toolkit had two areas supporting self-monitoring: the tracker and symptoms review. Some participants and carers tracked their symptoms, medication and activities to gain more information about them and to have a record for HCP appointments.

*So, what happened was, it went from [the supporter] going through it with me and us filling bits in, or me filling it in with him knowing what was going on, to then using it as sort of an emergency kit like with the daily trackers and the weekly trackers, I then took over. And it enabled me to discuss with the GP what was going on.* Carer of Participant 34, Male 71

Some participants did not use the tracker, mainly due to competing demands for time and difficulties understanding how to use it.

*No. I think the intention was that I should use the tracker in between conversations but I failed to do that. Just getting from day-to-day in life seems to take all the focus I can muster.* Participant 28, Female 72

The symptom tracker was helpful for participants to monitor their symptoms, especially as they became more severe, and linked participants to relevant information pages to help them manage.

*It has helped me think about that and try and evaluate what was happening.* Participant 14, Male 75

Other participants did not use the symptom tracker, as they could not see the benefit, as no changes in symptoms occurred during the intervention period, but felt it would be useful later.

*I can see that being very handy over time. Just to go back and check, you know, check the dates when you were describing various symptoms.* Participant 24, Female 55

#### 4.6.2. Changing or Maintaining Positive Behaviours

The ‘My Wellbeing' section of the toolkit encouraged participants to identify areas of their life they would like to maintain or improve and then set plans to work towards this. It aimed to support participants to develop problem-solving skills and develop their own solutions as new problems or concerns arose.

*It was very helpful in that regard, in terms of… ways in which it helped you look at your situation, ways in which it helped you look at what you might do. What things were happening to you and what you could do yourself about them*. Participant 14, Male, 75

Participants discussed specific behaviours that had changed through use of the toolkit or improvements they had seen in themselves. Some were linked to specific sections of the toolkit, such as the wellbeing section or the information section, whereas others were about more general toolkit use. For example, one person described how the toolkit helped them become more organised in their PD management.

*It has changed in a way. Like how I take the medication now, because of the toolkit. The change is how, in a broader framework, how I used to look at Parkinson's and how, you know, organised and how things can be by looking at that. I wasn't much of a time, timely, like I wouldn't take it much seriously. But with this, like, I can see where I stand, how I was before and how I can streamline to see that I do my things accordingly. In a way, it's organised my life a little bit, much better than what it was before.* Participant 03, Male 48

In general, the toolkit appeared to improve people's ability to self-manage their PD regardless of their previous experience of self-management.

*Because I've had stuff on my voice, for example, it's clear to me that my voice exercises should… play a more important part. The same goes for some other things. I was just stretching before we came on here, I need to spend more stretching. You get terribly stiff with Parkinson's, it kind of locks you into positions almost. And the… I think… I was almost halfway there with what your project is about because I think if it's about managing oneself and the situation.* Participant 14, Male 75

Participants discussed their motivation for using the toolkit and engaging with their plans to maintain their current abilities to enable them to live a life they valued. Some participants self-motivated themselves by reminding them of the benefits of doing the task.

*I think [Pauses] one's got to be reasonably hard on oneself with something like Parkinson's. Do the exercises, do the stretching, go out again. You know, I do sometimes get extremely tired and have to go and sit down almost. But often, I'm doing things when I'm pretty tired. I think you do have to push yourself a bit with something like Parkinson's, otherwise the tendency is just to sit down and not do much. I felt better for it.* Participant 14, Male 75

A small minority of participants chose not to set goals, as they were already proactive.

*I think, if I remember rightly, my goal setting was just to carry on doing what I was doing and I would have carried on doing that if I had done sessions or I hadn't done sessions, you know. I sort of make sure I do as much exercise as I can. So, I didn't set any goals which I wasn't doing already, really.* Participant 11, Male 60

#### 4.6.3. Context and Complexity

Most participants used the toolkit as a resource to access as and when it was needed or encountered a problem with their PD. This way of using the toolkit seemed to fit into people's lives more successfully.

*Well, I've been stop and go, I haven't been very continuously inside it. But once I've seen the features, I've been in to see the features and then occasionally when I thought of updating it, then I used it. Or if I needed anything to access the research part or just to help, I didn't have anything to do, I just wanted to go into the research part and see what's going on. So then, that time I used the toolkit, yes.* Participant 03, Male 48

One carer stated the reason they were not involved in the sessions and using the toolkit as much as they would have liked was due to a lack of time due to competing caring demands.

*I haven't really got the time to sit down now. We did in the beginning but it's … Yes, my time is taken up with looking after him.* Carer of Participant 05, Male 78

Others were concerned that they had to rely on other people to support them with their goals, and therefore, this hindered them at times.

*I can't go [out] by myself, so I have to have somebody with me. So, yes, it's… it's impinging on everybody's life. People don't mind doing it once or twice but, you know, it becomes every week, it becomes a drag.* Participant 26, Female 72

Some participants had specific symptoms from PD or other conditions that hindered how they accessed and engaged with the toolkit.

*Because of my eyesight, I've got macular degeneration. Reading was almost impossible. Yes, I got large text, when they said to go to large text. Well, it may be large but it's not large enough.* Participant 05, Male 78

*Well, we were exploring the toolkit and what it could do and where I could put things, which I then followed up and tried to do but my memory is not that great and I could never remember what she said, so I would just experiment and put things in different places and then not remember where I'd put them.* Participant 12, Female 66

When considering what stage of diagnosis would be best to access the toolkit, there were differing views. One participant said they felt they were too early in the stage of the disease to use the toolkit.

*I haven't used [the toolkit] … my attitude is when I need to think about Parkinson's, I'll think about… It's not something that I fill my waiting days with, sort of thinking about Parkinson's… I just prefer to just bury my head in the sand really and get on and while it's good on a day-to-day basis, great. But, you know, I think my attitude has always been that when my condition worsens and I do feel I need some help, then I'll go further into it then.* Participant 11, Male 60

Most participants looked back to when they were diagnosed and felt the toolkit would have been a helpful guide for them and would have reduced wasted time looking for the right information on their PD.

*This information 11 or 12 years ago would have been extremely helpful. But for the last all these years we've been dealing with things ourselves, you know, and finding out about things after the event. Whereas with this toolkit that you've developed, it's going to be wonderful for people who find out if they've got Parkinson's at early stages, it's going to be absolutely incredible. All these things that are in the folder, we've had to go the long way around with trying to find out what we should and shouldn't do and what we can and cannot do.* Participant 05, Male 78

## 5. Discussion

### 5.1. Summary of Findings

In this study we tested the feasibility and acceptability of a self-management toolkit for people with PD and their carers, ahead of a large-scale RCT. We successfully recruited the target population within a predefined timeline, maintained engagement with the intervention and completed sufficient follow-up, with minimal missing data and no intervention-related adverse events.

The intervention was delivered as intended for an average of 93% of intervention components, as measured using fidelity checklists, highlighting excellent agreement of intervention fidelity. For engagement with the intervention, 89% were engaged, attending at least three out of the 4 available sessions with the ‘*Live Well*' supporter. This met our predefined threshold of 80%.

Through quantitative questionnaire data, at least 70% of participants found six of the toolkit sections to be helpful, suggesting acceptability. The tracker and calendar were found to be the least helpful, by 53% and 47%, respectively. Participants who used the symptom tracker found this useful to see how things were changing over time and to inform conversations with HCPs. No-one interviewed had had the opportunity to do this , due to a lack of HCP appointments during the study window, perhaps due to COVID-19-related delays. A previous systematic review found patients who tracked before consultations felt their interactions and discussions were improved if the HCPs were receptive and encouraging of this behaviour [14]. Following up on healthcare consultations 3–15 months after receiving an educational self-management intervention in Sweden, the strategies and techniques taught were being used in everyday life and discussed with HCPs [39], highlighting the long-term impact that self-management and tracking can have on interactions with HCPs.

Barriers to tracking were a lack of motivation, a lack of understanding of how to use the feature in the toolkit, and a lack of perceived benefit due to the slow deterioration associated with PD. In other interview studies with people with PD, barriers to tracking included digital literacy, lack of perceived usefulness of tracking and difficulty describing symptoms [40, 41]. More general barriers to toolkit use were competing demands of their health and their time. Using the toolkit as and when it was needed, rather than at fixed timepoints, seemed to work well for most participants. However, for those with lower motivation, having a regular reminder to use the toolkit on a weekly or monthly basis was essential. Reminders have been suggested for other ways of supporting people with PD to complete health-related behaviours, like adhering to medication regimes and participating in physical activity [39, 42].

The sessions with the supporter were deemed to be the most helpful, by 94% of participants, with interview participants highlighting the importance of having someone to listen to them and guide them through which parts of the toolkit were most useful for their specific needs. This reflects the findings of a recent systematic review of the importance of facilitation to increase uptake of digital interventions by primary care patients [43]. The toolkit was found to be a reliable, comprehensive resource for people with PD and their carers to find information and empower them to be able to self-manage. The review also highlighted the importance of the perceived usefulness, trust in the resource and staff motivation to promote engagement with digital interventions [43]. Some suggestions for improvement were to present the information in different formats, such as videos, to make it more accessible and less overwhelming.

Setting wellbeing priorities (goals) was self-reported in 63% of participants, and 63% of these felt they had made much progress towards their goal. Participants set person-centred goals that were important for them, often around exercise and mobility and medication adherence, aligning with the common goal type categories identified in a systematic review [14]. However, a recent scoping review identified that physical activity levels achieved by people with PD often do not meet recommendations or intervention objectives [44], highlighting the challenge of engaging people with PD to participate in physical activity goal setting and achievement. An RCT, testing an exercise and self-management intervention in Australia, is ongoing and will provide additional insights into how combining physical activity and self-management can impact physical activity, quality of life and self-efficacy outcomes [45].

Those already proactively working on their wellbeing chose to maintain what they were already doing, but this approach made them less likely to record their activities in the tracker, again highlighting the need for perceived usefulness of tracking to motivate people to engage in this behaviour [41]. Some felt that, due to the many years between diagnosis and accessing the toolkit, they had already sourced most of the information themselves and put steps into place to self-manage their PD. Most recommended providing access to the toolkit in the first few years of PD diagnosis. This is supported by research conducted in Sweden, which highlighted that people with PD often seek information about the disease online and that those who read and know more about the condition feel happier with the care they receive, irrespective of the amount of help they receive from HCPs [46]. However, whilst the intervention may be more effective if provided from diagnosis, the aim of the development of this toolkit is to support self-management for patients at all stages and be applicable in a range of different settings.

### 5.2. Modifications to the Intervention and Study Procedures

Based on the feedback and recommendations in the qualitative feedback, some modifications to the UCL *Live Well with Parkinson's* toolkit were made. The main change to the toolkit was the addition of videos, with audio, to each of the information pages to make them more accessible and to break up the text.

The main change to the trial procedures for the full RCT was changing the primary outcome measure from the MDS-UPDRS parts I and II to the PDQ-39 total score. This was based on the qualitative findings from this feasibility study, suggesting that a change in quality of life is more attainable, whereas changes in disease progression are unlikely. The fidelity checklists were updated to reflect the sessions more accurately and to make a distinction between the specific behaviour change techniques.

### 5.3. Limitations

For this feasibility study, participants were recruited from London only, and the intervention was delivered by one supporter, and so the findings from this feasibility study may lack generalisability. These aspects will be addressed as part of the large-scale RCT and its internal pilot, where participants will be recruited from across the country to ensure maximum diversity and generalisability of the results.

When testing fidelity and engagement, only supporter-completed checklists and attendance at sessions were used. From the digital toolkit, the data analytics have not been analysed as part of this feasibility study and would have provided more in-depth understanding of participants' independent engagement with the intervention. As part of the RCT, a full analysis of digital analytics will be completed to understand this further in a larger sample.

This feasibility study was conducted during a COVID-19 lockdown and many of our participants reported that the isolation and lack of healthcare services available to them were having a large impact on them. Therefore, some of our findings may be more related to this context, as opposed to the intervention itself.

## 6. Conclusions

We found that a self-management toolkit for people with PD and their carers was acceptable to support them to manage their symptoms and to set and maintain their wellbeing priorities. We recruited and retained participants in the study at an acceptable rate, with minimal loss to follow-up and missing data. As all the feasibility criteria were met, with the above modifications we have consequently progressed to the PD-Care: UCL *Live Well with Parkinson's* RCT, in which the clinical and cost-effectiveness of the toolkit is currently being tested [20].

## Figures and Tables

**Table 1:** Participants' characteristics of the 35 participants recruited into the feasibility study.

Characteristic	*N* (%)
Gender	
Female	11 (31.4%)
Male	24 (68.6%)
Age (years)—mean (SD)	69.1 (10.7)
Ethnicity	
White (British)	26 (74.3%)
Mixed (British/other)	1 (2.9%)
Asian (British/other)	7 (20.0%)
Black (British/other)	1 (2.9%)
Marital status	
Single/unmarried	2 (5.7%)
Married	23 (65.7%)
Cohabiting	2 (5.7%)
Divorced	4 (11.4%)
Widow/widower	4 (11.4%)
Living situation	
Spouse/life-partner	14 (40.0%)
Family	9 (25.7%)
Alone	6 (17.1%)
Other	6 (17.1%)
Location	
Major conurbation	1 (2.9%)
City	20 (57.1%)
Town	10 (28.6%)
Village	4 (11.4%)
Age left full-time education—mean (SD)	20.6 (5.4)
Indices of multiple deprivation	
1–3 (most deprived)	7 (20.0%)
4–6	10 (28.6%)
7–10 (least deprived)	18 (51.4%)

**Table 2:** Mean (SD) scores for the outcome measures collected at baseline and 3-month follow-up.

Measure	Baseline (*n* = 35)	Follow-up (*n* = 31)
	Mean (SD)	
MDS-UPDRS part I	12.9 (8.8)	12.4 (8.2)
MDS-UPDRS part II	15.1 (12.1)	14.9 (11.5)
MDS-UPDRS part IV	6.4 (5.2)	7.0 (4.3)
MDS-NMS	142.7 (103.1)	108.7 (78.8)
PDQ-39	27.4 (21.7)	27.7 (22.6)
GHQ-12	12.7 (5.9)	13.1 (6.3)
Self-efficacy for managing chronic disease	38.5 (13.6)	40.0 (13.9)
ICECAP-O	0.5 (0.3)	0.5 (0.3)
TMoCA	18.1 (2.6)	N/A
PAM-13	61.5 (13.3)	N/A

**Table 3:** Number and percentage of participants rating the corresponding toolkit section [20, 21] as helpful or very helpful on the feedback questionnaire.

Section	*N*	Helpful or very helpful
About me	27	22 (81%)
My health	29	23 (79%)
My symptoms	30	21 (70%)
My wellbeing	29	21 (72%)
My tracker	29	17 (59%)
Calendar	29	14 (48%)
Information	30	22 (73%)
Sessions	30	28 (93%)

**Table 4:** Demographic characteristics of the 14 participants interviewed.

Characteristic	*N* (%)
Gender	
Female	6 (42.9%)
Male	8 (57.1%)
Age (years)—mean (SD)	67.6 (10.0)
Ethnicity	
White (British)	10 (71.4%)
Asian (British/other)	3 (21.4%)
Black (British/other)	1 (7.1%)
Age left full-time education—mean (SD)	20.6 (5.4)
Indices of multiple deprivation	
1–3 (most deprived)	5 (35.7%)
4–6	4 (28.6%)
7–10 (least deprived)	5 (35.7%)
Toolkit type	
Paper	1 (7.1%)
Online	11 (7.9%)
Both	2 (14.3%)

## Data Availability

The data that support the findings of this study are available from the corresponding author upon reasonable request.

## Supporting Information

Supporting Figure 1: UCL *Live Well with Parkinson's* toolkit logic model.
