# Supplementary material for: Feasibility and Acceptability of Intervention and Trial Procedures of the UCL Live Well With Parkinson's Self-Management Toolkit
Source: Parkinsons Dis. 2025 Aug 21;2025:2804226. doi: 10.1155/padi/2804226 (PMC12393956; doi:10.1155/padi/2804226)
Supplement: Supporting Information — Additional supporting information can be found online in the Supporting Information section. [file 2804226.f1.pptx]

## Slide 1
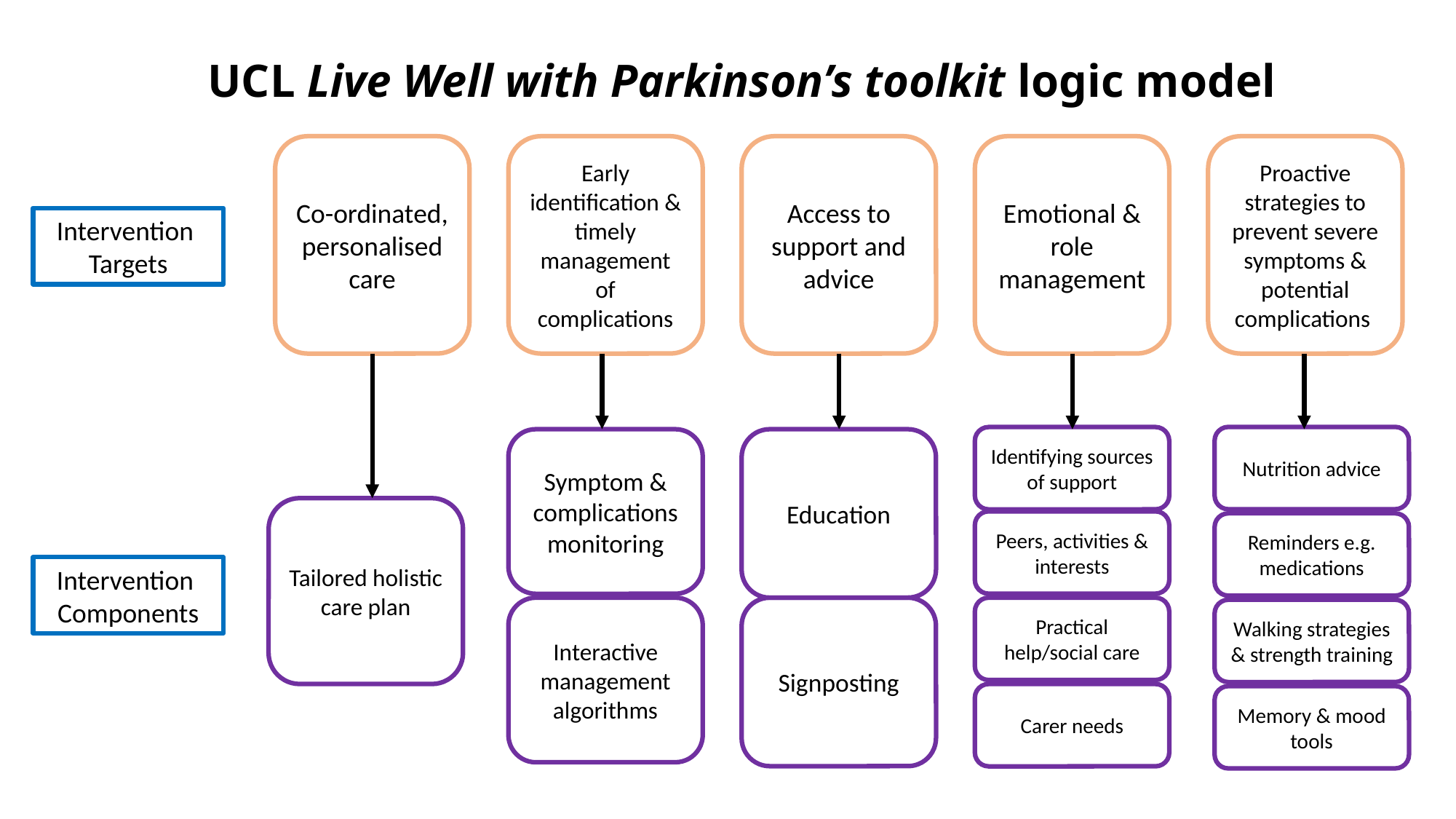

# UCL Live Well with Parkinson’s toolkit logic model
Co-ordinated, personalised care
Access to support and advice
Emotional & role management
Proactive strategies to prevent severe symptoms & potential complications
Early identification & timely management of complications
Intervention
Targets
Nutrition advice
Reminders e.g. medications
Walking strategies & strength training
Memory & mood tools
Identifying sources of support
Peers, activities & interests
Practical help/social care
Carer needs
Education
Signposting
Symptom & complications monitoring
Interactive management algorithms
Tailored holistic care plan
Intervention
Components
